# Acute, high dose ethylene glycol administration produces no neuroinflammation, no abnormal brain or liver histology, significant urinary oxalate excretion, and minimal nephrotoxicity in male CD-1 mice

**DOI:** 10.1016/j.toxrep.2026.102342

**Published:** 2026-09-15

**Authors:** Greg M. Landry, Thomas Q. Dong, James Boksanski

**Affiliations:** Massachusetts College of Pharmacy and Health Sciences, School of Pharmacy, Department of Pharmaceutical Sciences, Boston, MA, 02115, United States

**Keywords:** Ethylene glycol, Species sensitivity, Neurotoxicity, Acute kidney injury, Calcium oxalate

## Abstract

The hallmarks of ethylene glycol (EG) toxicity are very well characterized in rat and female mouse models, and includes glycolate-mediated metabolic acidosis with calcium oxalate monohydrate (COM) target-organ accumulation. However, high dose, acute EG target organ toxicity has yet to be assessed in male CD-1 mice. Therefore, our studies utilize 9-week-old, male CD-1 mice to assess EG toxicity at 3 concentrations (3, 6, and 12 g/kg) in the mouse for 24, 48, and 72 h. Results indicate that 6 and 12 g/kg EG induce a 2.5-fold increase in serum creatinine 72h-post gavage with minor kidney damage, and no COM crystal tissue deposition with majority of oxalate being excreted in the urine. Oxalate alone (4 and 10 mM) induces a 27% and 31% decrease in ATP, respectively after 6 and 12 h in mouse proximal tubule cells *in vitro*. Additionally, only the 12 g/kg EG dose induced significant metabolic acidosis at 24 h, but not at 48 or 72 h. Furthermore, at these EG doses there was no gross brain tissue injury or neuroinflammation. These results imply that even at significantly higher EG doses, male CD-1 mice are insensitive to EG-induced target organ damage due to high urinary oxalate excretion.

## Highlights

• Ethylene glycol is only minimally nephrotoxic to male CD-1 mice at high doses.
• Male CD-1 mice have no target organ COM accumulation decreasing EG-induced toxic effects.
• High dose EG induces no neuroinflammation or abnormal brain histology in male CD-1 mice.
• Oxalate alone is nephrotoxic in mouse proximal tubule cells *in vitro*.

## Graphical Abstract

**Figure:**
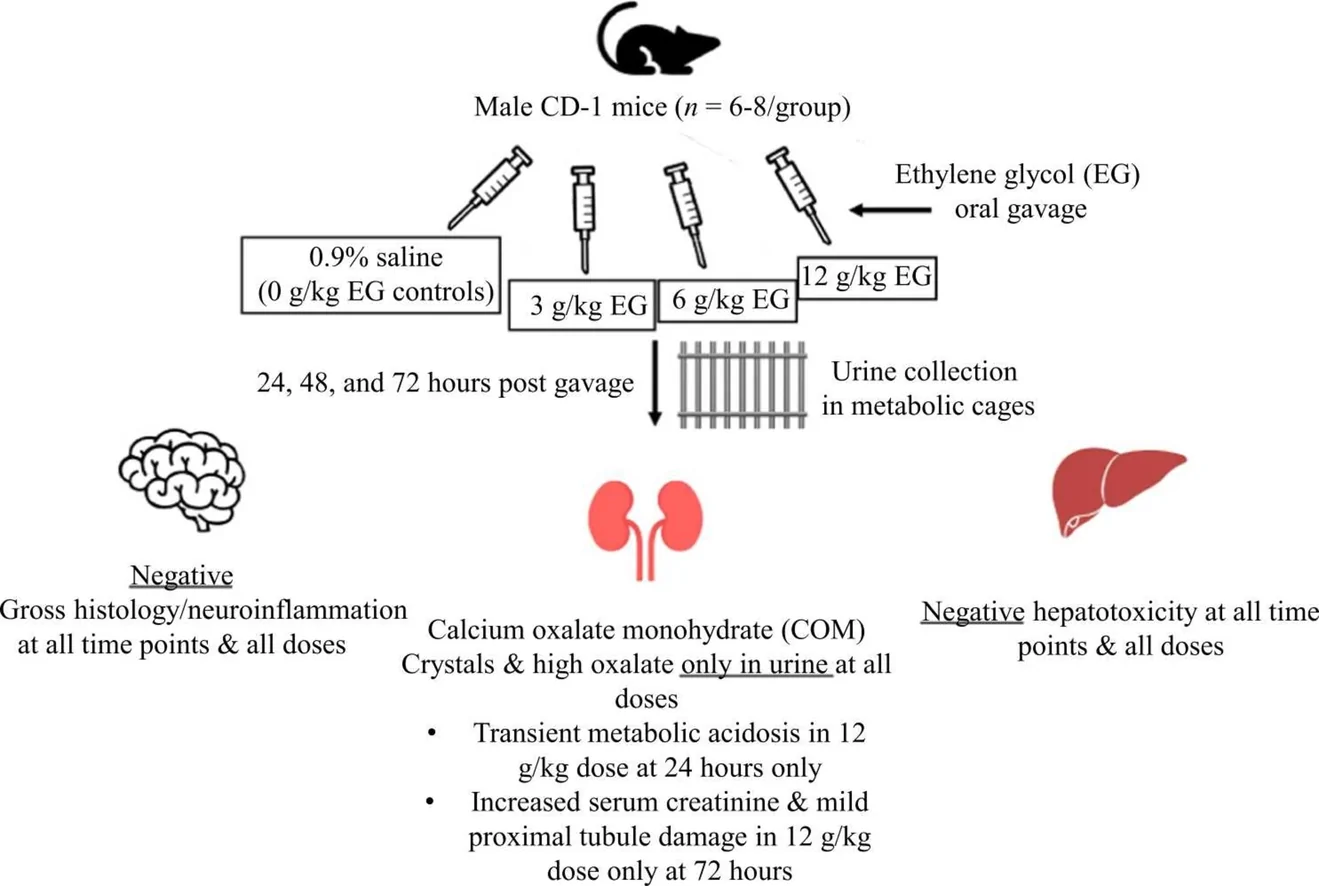

## 1 Introduction

Ethylene glycol (EG, CAS #107–21–1) is primarily utilized industrially in the synthesis of polyethylene terephthalate resins, unsaturated polyester resins, and polyester fibers. Better known uses are as the major constituent in antifreeze, deicing fluid, heat transfer fluids, industrial coolants, and hydraulic fluids. The multitude of uses increases the risk for potential occupational and consumer exposure Ingestion of EG, whether accidental or intentional, remains a significant toxicological concern due to its high mammalian toxicity [1]. The metabolism of ethylene glycol is central to both the acute and chronic target organ toxicity.

Consumption of significant quantities of EG (approximately(~) > 100 mL in adult humans) typically progresses from an initial CNS toxicity phase (~0.5–12 h) to a cardiopulmonary phase concurrent with metabolic acidosis (~12–24 h), and finally renal toxicity (1–3 days) [2]. Rapid gastrointestinal absorption and extensive hepatic metabolism to glycolic acid is responsible for the metabolic acidosis observed post-EG ingestion. Oxalate, which precipitates as calcium oxalate monohydrate (COM)accumulates in various tissues, specifically the kidney, and can result in acute kidney injury (AKI). [[3], [4], [5], [6], [7], [8], [9], [10]]. Despite decades of experimental studies, the foundational understanding of acute EG toxicity is derived from non-murine models, specifically rats. These studies focused on EG-induced AKI, cardiopulmonary toxicity, as well as developmental and reproductive toxicity. Additionally, a recent, acute, nephrotoxicity study utilizing Deutschland Denken Yoken male mice characterized an oral EG LD_50_ (12–15 g/kg) after only 24 h post-EG administration with no assessment of metabolic acidosis or neurotoxicity [11].

Beyond the one acute toxicity study in mice, a significant body of literature has evaluated EG-induced developmental toxicity across species including mice, rats, and rabbits; whereby, the vast majority of EG studies in mice have been to assess developmental and reproductive toxicity only [[12], [13], [14], [15], [16], [17], [18], [19]]. Early foundational work examined reproductive and developmental outcomes in female CD-1 mice exposed to EG via drinking water in a continuous breeding protocol [16]. These studies demonstrated dose-related effects on reproductive performance and offspring viability. This study provided some of the earliest evidence for EG-induced developmental toxicity in murine models. Subsequent investigations expanded on these findings documenting craniofacial defects, neural tube closure abnormalities, and axial skeletal dysplasia following gestational exposure [12,17]. More recently, inhalation studies in mice and rats have demonstrated decreased fetal weight, reduced implantation viability, and increased incidences of skeletal and visceral malformations at concentrations ≥ 1000 mg/m³ during gestational days 6–15 [20,21]. Developmental toxicity appears to be closely linked to metabolic saturation at the NOAEL and LOAEL for developmental toxicity in mice and rats at 0.5 g/kg/day and 0.15 g/kg/day, respectively [14]. This threshold indicates a nonlinear dose-response relationship observed with other similar glycols [14,22], and highlights the relevance of high-dose experimental conditions to observe outcomes.

While EG toxicity characterization has predominantly focused on renal, developmental, and cardiopulmonary effects, the alcohol-like properties of EG induce CNS depression and intoxication with altered mental status likely attributable to high circulating levels of unmetabolized EG [2]. Severe CNS effects observed post-EG ingestion, such as cerebral edema, seizures, and coma [23] likely occur post-AKI, but can be difficult to delineate an ultimate cause. To our knowledge, there are no studies directly assessing brain histopathology and inflammation in male CD-1 mice treated with EG with or without AKI. Frantz et al., 1996 reported ^14^C-labeled EG distribution being lowest in the brain (21.77 ± 2.62 µg/g tissue) of female CD-1 mice after oral administration of 1000 mg/kg EG at 96 h post gavage with the highest being the liver (119.79 ± 24.01 µg/g tissue), followed by kidney (71.70 ± 4.54 µg/g tissue), and then lung (62.92 ± 26.92 µg/g tissue) [24]. These data have preliminary indications that EG likely does not reach the brain at significant enough concentrations to be fully responsible for CNS effects observed clinically without concomitant AKI.

Previous literature assessing EG-induced kidney toxicity in male mice utilized the B6C3F_1_, Black Swiss, and C57BL/6 strains [[25], [26], [27]] providing a reasonable gap in the literature to assess EG-induced kidney, brain, and liver toxicity in the male CD-1 mouse commonly and historically utilized only for teratogenic assessment of EG [16,20,21]. We hypothesize that male CD-1 mice will exhibit the same or similar EG-induced target organ toxicities as their male strain counterparts. Therefore, the purpose of this acute, high dose mouse study was to further characterize this dose paradigm in male CD-1 mice at later time points including 24, 48, and 72 h and higher EG concentrations (3, 6, or 12 g/kg). These concentrations were chosen to mimic large, acute doses as there are a significant number of case reports indicating ingestion amounts ranging from ½-entire bottles to 3–4 liters in humans [[28], [29], [30], [31], [32], [33], [34], [35], [36], [37], [38], [39], [40], [41], [42], [43], [44]]EG-mediated target organ (kidney, brain, and liver) effects were assessed, and whether or not these doses at these time points induced changes in kidney, liver, or brain pathology or inflammation.

## 2 Materials and methods

### 2.1 ARRIVE guidelines

All methods are reported in accordance with Animal Research: Reporting of *In Vivo* Experiments (ARRIVE) guidelines and regulations.

### 2.2 Animal protocol

Nine-week-old, adult male CD-1 mice weighing an average of 35 g/mouse (range: 30–39 g) were randomly assigned without bias by the investigators with no exclusions to treatment groups: saline control (0 g/kg; n = 6 in the 24 and 72 h groups, n = 8 in the 48 h group), 3 g/kg EG (n = 6 in the 24 and 72 h groups, n = 8 in the 48 h group), 6 g/kg EG (n = 6 in the 24 and 72 h groups, n = 8 in the 48 h group), and 12 g/kg EG (n = 6 in the 24 and 72 h groups, n = 8 in the 48 h group). Sample size was chosen based on previous literature dosing ethylene glycol in rodents, and in accordance with previous power analyses. Animals were fasted for 12 h prior to dosing and then administered 0.9% saline or EG by oral gavage at time 0, followed by housing in mouse metabolic chambers for 24, 48, or 72 h urine collections, which is sufficient to obtain measurable amounts of sample in mice. EG was undiluted, and was administered via oral gavage based on individual mouse weight. Mice receiving a dose of 3 g/kg EG at each time point received an average gavage volume of 0.088 mL for the 24 h group, 0.096 mL for the 48 h group, and 0.095 mL for the 72 h group. Mice receiving a dose of 6 g/kg EG at each time point received an average gavage volume of 0.177 mL for the 24 h group, 0.189 mL for the 48 h group, 0.211 mL for the 72 h group. Mice receiving a dose of 12 g/kg EG at each time point received an average gavage volume of 0.323 mL for the 24 h group, 0.384 mL for the 48 h group, and 0.376 mL for the 72 h group. Standard animal facility conditions (temperature 25 ± 2 °C; 12:12 h light: dark cycle) were maintained, and mice had ad libitum access to food (standard chow) and water except during fasting periods. The animal protocols were ethically approved by the Institutional Animal Care and Use Committee (Massachusetts College of Pharmacy & Health Sciences) and were in accordance with the National Institutes of Health “Guide for the Care and Use of Laboratory Animals.”

### 2.3 Animal observations

Animals were monitored regularly for morbidity and behavioral changes (e.g., food and water intake, responsiveness to stimuli).

### 2.4 Urine collection and analysis

Urine was collected into tubes at 6, 12, 24, 36, 48, 60, and 72 h (as applicable by study design). Collection chambers were rinsed between time points. Urine volume and pH were recorded immediately; samples were transferred to microcentrifuge tubes and stored at −20 °C for analysis. Urinary oxalate concentrations were measured using a commercially available colorimetric Oxalate Assay Kit (MAK315; Sigma-Aldrich, St. Louis, MO, USA) following the manufacturer’s protocol. Samples were thawed and centrifuged at 1000 rpm for 10 min to remove insoluble material, and the resulting supernatants were collected for analysis. Oxalate concentrations were determined using the oxalate oxidase-based reaction with absorbance measured at 595 nm (Synergy HT microplate reader, BioTek, Winooski, VT). Oxalate concentrations were calculated using supplied oxalate internal standard according to the manufacturer’s protocol and reported as µmol/L.

### 2.5 Blood collection and analysis

Mice were anesthetized at either 24, 48, or 72 h using isoflurane. Whole blood was collected from the cheek vein into heparinized tubes and analyzed for pH, pCO₂, Hct, tHb, Na, K, Cl, ionized Ca and Mg, glucose, serum creatinine, blood urea nitrogen (BUN), total CO₂, HCO₃⁻, oxygen capacity, anion gap (K), and osmolality using a blood gas analyzer (Stat Profile Prime Plus® VET Critical Care Analyzer, Waltham, MA). Additional blood was centrifuged at 20,000 ×g for 10 min to isolate plasma, which was stored at −80 °C for further analysis.

### 2.6 Tissue collection and histopathology

After anesthetizing (1–3% isoflurane induction with 4% maintenance) the mice, kidneys, brain, and whole liver from all treatment groups were collected and weighed for histopathological analysis. One whole kidney and one lobe of the liver and brain were immediately flash frozen. The remaining liver, kidney and brain were placed in 10% neutral buffered formalin. All organs were sent to Histowiz for slicing, staining, and scoring. Tissues from brain, kidney, liver were stored in 10% neutral buffered formalin, processed, paraffin embedded, sectioned, placed on glass slides, stained with hematoxylin and eosin (H&E), digitally scanned on a Aperio GT450 scanner (Leica Biosystems, Inc) creating whole slide images (WSIs), and uploaded to the Histowiz cloud platform. Slides were evaluated by a blinded senior research pathologist with no prior knowledge of treatment groups or experimental design. WSIs were reviewed in entirety, annotated online and control types were not designated to prevent bias. Brain WSIs were assessed for necrosis, hemorrhage, inflammation, degeneration, fibrosis, microcalcification, and vacuolation. Liver WSIs were assessed for steatosis, inflammation, necrosis, fibrosis, EMH, ballooning degeneration, glycogen accumulation, cytoplasmic vacuoles, single cell apoptosis, and hemorrhage. Kidney WSIs were assessed for necrosis, hemorrhage, inflammation, tubular basophilia, tubular vacuolation, fibrosis, microcalcification, cystic change, tubular dilatation, and glomerular change. Severity scores were assigned as follows:

**Table:** 

Severity	Proportion of tissue affected	Grade	Quantifiable Finding
None	0%	0	0
Marginal or minimal	1–10%	1	1–2 foci
Slight or few	11–25%	2	3–6 foci
Moderate or several	26–50%	3	7–12 foci
Marked or many	51–75%	4	> 12 foci
Severe	> 76%	5	Diffuse

### 2.7 Measurement of brain TNF-α, IL-6, and Iba-1 in EG-treated mice

To assess EG-induced brain inflammation, flash frozen brain tissue from all groups collected above were coronally sliced and vertically sectioned at the medial/temporal region to isolate the hippocampus. Sections were immediately transferred into Eppendorf tubes containing lysis buffer on ice containing 150 µL of 1X PBS, pH 7.4, 0.05% Triton-X-100, and protease inhibitor cocktail, and homogenized using sonication. TNF-α, IL-6, and Iba-1 expression levels were assessed using the mouse TNF-α ELISA and mouse IL-6 ELISA kits (Abcam, Waltham, AM) and the mouse AIF/Iba-1 ELISA kit (ThermoFisher Scientific, Waltham, MA) following manufacturer’s protocols.

### 2.8 Cell culture

Mouse TKPTS proximal tubule cells were maintained in DMEM/F-12 medium supplemented with 7% fetal bovine serum and 0.3 mL of 10 mg/mL insulin. Cells were incubated at 37 °C in 5% CO₂ and 90% humidity. For ATP measurements, cells were grown to confluence in opaque-walled, clear-bottom 96-well plates.

### 2.9 Measurement of mouse TKPTS proximal tubule cell viability

Mouse TKPTS proximal tubule cell viability following sodium oxalate exposure was used to evaluate the role of oxalate ion-induced proximal tubule toxicity. The CellTiter-Glo Luminescent Cell Viability Assay (Promega, Madison, WI), which quantifies cellular ATP as an indicator of metabolically active cells, was utilized. All treatments were prepared in a calcium-free ((-)Ca^2+^) buffer containing 124 mM choline chloride, 124 mM NaCl, 5.3 mM KCl, 1.0 mM MgCl2, 7 mM glucose, and 20 mM HEPES, adjusted to pH 7.4. TKPTS cells were exposed to increasing concentrations of sodium oxalate (0.2, 0.5, 1, 2, 4, or 10 mM) or (-)Ca^2+^ buffer controls for 1, 6, or 12 h. Earlier time points were assessed to decrease any off-target toxic effects from exposure to a calcium free environment for an extended time period. Calcium-free buffer without sodium oxalate served as the negative control, while 100 mM sodium oxalate served as the positive control for cytotoxicity. Following each exposure period, the CellTiter-Glo assay was performed according to the manufacturer’s instructions. Luminescence was measured using a microplate reader with the instrument gain set to 135. Cell viability was determined from luminescence values relative to the (-)Ca^2+^ buffer control.

### 2.10 Statistical analysis

**Fig. 1:**
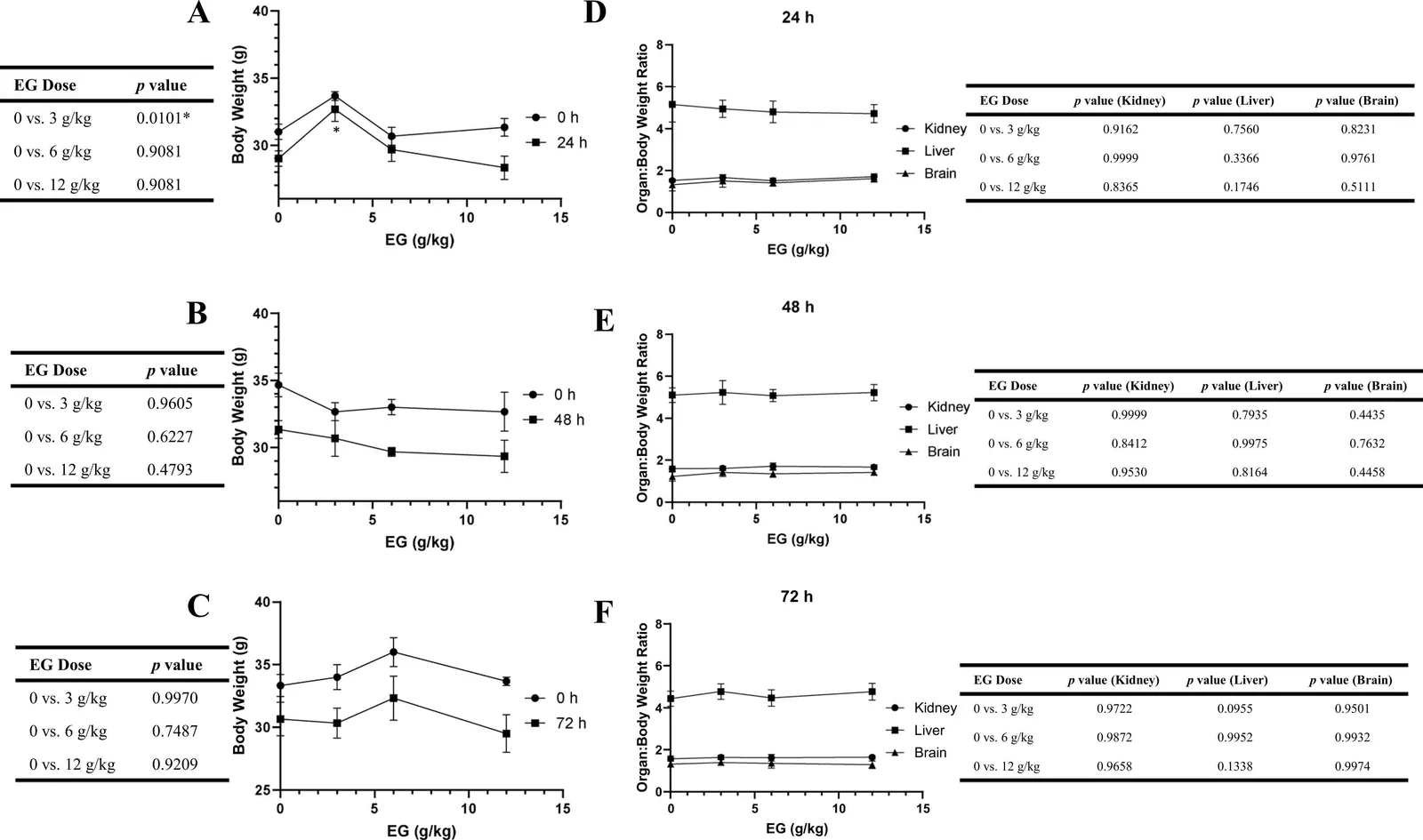
Acute ethylene glycol (3, 6, or 12 g/kg) administration at 24, 48, or 72 h in male CD-1 mice does not induce significant changes in overall body weight when compared to weights of mice at 0 h at each time point or when compared to 0 g/kg EG control animals (A-C) nor does it significantly affect kidney:, liver:, or brain: body weight ratios when compared to 0 g/kg EG control animals at all time points (D-F). Data is represented by mean ± S.E.M. (*n* = 6–8), and was analyzed using a two-way ANOVA followed by a Bonferroni’s *post-hoc* test; p < 0.05 would have indicated significance with individual *p* values indicated in the corresponding Tables.

**Fig. 2:**
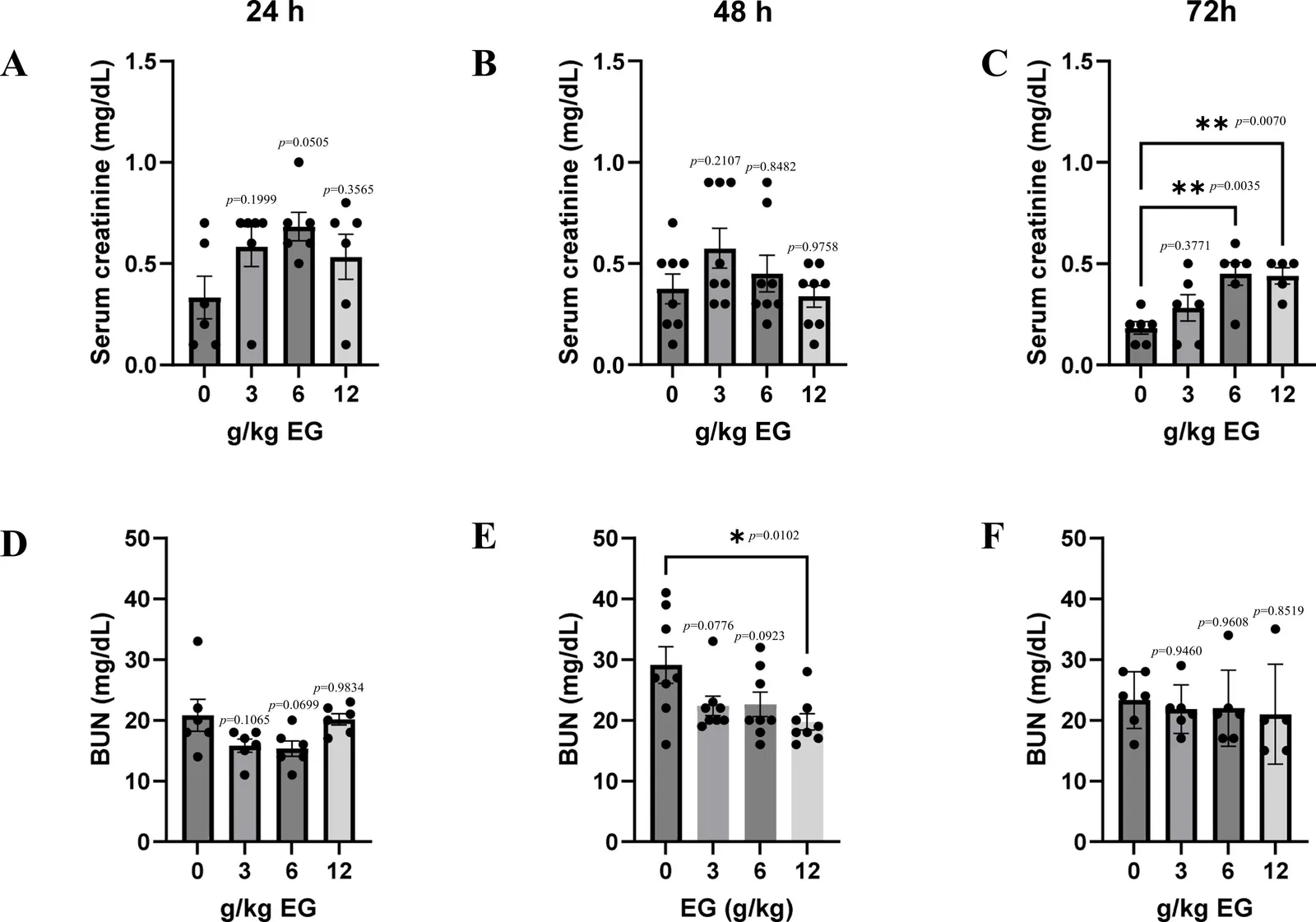
Acute ethylene glycol (EG) administration of 6 and 12 g/kg induce significant increases in serum creatinine at 72 h (C), but not at 24 or 48 h (A-B) in male CD-1 mice. 12 g/kg EG induces a significant decrease in blood urea nitrogen (BUN) at 48 h (B), but not at 24 or 72 h (D, F). Data is represented by mean ± S.E.M. (*n* = 6–8). Asterisks (*) indicate significant difference when compared to 0 g/kg EG control animals, and was analyzed using a one-way ANOVA followed by a Dunnett’s *post-hoc* test. *p < 0.05; **p < 0.01 with individual *p* values indicated above each EG treatment group.

Data were analyzed using GraphPad Prism 11 for Windows (GraphPad Software, La Jolla, CA). Data were analyzed either by one-way ANOVA followed by Dunnett’s *post-hoc* test or a two-way ANOVA followed by Tukey’s *post-hoc* test, as appropriate. Data in Fig. 1 was analyzed using a two-way ANOVA followed by a Tukey’s *post-hoc* test to delineate differences in two variables: body weight and organ weight across time and EG dose, respectively. Data in Fig. 2, Fig. 7 were analyzed using a one-way ANOVA followed by a Dunnett’s *post-hoc* test to delineate differences in the EG-treated animals compared to the control animals (0 g/kg). Data in Fig. 9 was also analyzed using a one-way ANOVA followed by a Dunnett’s *post-hoc* test to delineate differences between mouse TKPTS proximal tubule cells exposed to increasing concentrations of Na^+^ oxalate compared to control cells exposed to calcium free ((-)Ca^2+^) control buffer alone. Data is normally distributed within each group, and the variance of the dependent variable(s) are roughly equal between each group, as well necessitating the use of these statistical tools to test for significance. Data are presented as mean ± SEM, where *n* represents the number of animals as biological duplicates in the *in vivo* studies. Data are presented as mean ± SEM, where *n* represents 6 different passage numbers of the immortalized mouse TKPTS proximal tubule cell line and statistical significance for all experiments was defined as p < *0.05*.

## 3 Results

### 3.1 Ethylene glycol does not significantly decrease body or affect individual organ weight in male CD-1 mice even at high doses

Animals did not exhibit significant decreases in body weight at 24, 48, or 72 h when compared to 0 h or when compared to 0 g/kg EG controls **(**Fig. 1**A-C)**. There was a slight, yet significant increase in average body weight only at 24 h in animals that received 3 g/kg EG **(**Fig. 1**A)**. Additionally, kidney, liver, and brain to body weight ratios did not significantly increase or decrease when compared to 0 g/kg EG controls **(**Fig. 1**D-F)**.

### 3.2 Ethylene glycol induces metabolic acidosis in male CD-1 mice at 24 h with resolution at 48 and 72 h

Only a significantly high dose of ethylene glycol (12 g/kg) induced metabolic acidosis in CD-1 male mice after 24 h of exposure **(**Table 1**)**. This was indicated by significant decreases in blood pH, bicarbonate (HCO_3_^-^), and total CO_2_ with subsequent significant increases in the anion gap and ionized Ca^2+^ in the plasma when compared to 0 g/kg treated mice at each time point **(**Table 1**).** These aforementioned parameters all return to baseline with no significant differences by 48 or 72 h post-EG exposure.

**Table 1:** Markers of Metabolic Acidosis in EG treated male CD-1 mice.

Time point:	24 h	48 h	72 h
**EG Dose**	**Blood pH**	**Blood pH**	**Blood pH**
0 g/kg	7.28 ± 0.03	7.27 ± 0.03	7.30 ± 0.03
3 g/kg	7.35 ± 0.03	7.35 ± 0.01	7.31 ± 0.03
6 g/kg	7.31 ± 0.04	7.30 ± 0.02	7.35 ± 0.01
12 g/kg	7.08 ± 0.03***	7.32 ± 0.02	7.33 ± 0.02
**EG Dose**	**HCO**_**3**_^**-**^**(mmol/L)**	**HCO**_**3**_^**-**^**(mmol/L)**	**HCO**_**3**_^**-**^**(mmol/L)**
0 g/kg	21.1 ± 0.8	21.7 ± 1.3	21.5 ± 0.8
3 g/kg	22.0 ± 2.1	23.3 ± 1.1	22.2 ± 2.1
6 g/kg	19.9 ± 1.0	24.8 ± 2.0	21.7 ± 1.0
12 g/kg	10.8 ± 1.5***	25.0 ± 1.4	26.2 ± 2.3
**EG Dose**	**CO**_**2**_**(mmol/L)**	**CO**_**2**_**(mmol/L)**	**CO**_**2**_**(mmol/L)**
0 g/kg	22.5 ± 0.9	17.1 ± 3.8	22.8 ± 0.9
3 g/kg	23.3 ± 2.2	18.0 ± 3.9	23.5 ± 2.1
6 g/kg	21.1 ± 1.0	18.2 ± 4.1	23.0 ± 1.1
12 g/kg	11.9 ± 1.6***	19.7 ± 4.1	27.7 ± 2.4
**EG Dose**	**Anion Gap**	**Anion Gap**	**Anion Gap**
0 g/kg	15.4 ± 1.8	20.3 ± 0.8	20.2 ± 0.9
3 g/kg	14.8 ± 1.8	18.6 ± 0.8	18.4 ± 0.7
6 g/kg	18.6 ± 2.9	17.4 ± 1.2	19.4 ± 0.6
12 g/kg	28.2 ± 1.7***	16.7 ± 0.9	17.8 ± 1.3
**EG Dose**	**iCa**^**2+**^**(mmol/L)**	**iCa**^**2+**^**(mmol/L)**	**iCa**^**2+**^**(mmol/L)**
0 g/kg	1.25 ± 0.02	1.29 ± 0.01	1.28 ± 0.01
3 g/kg	1.23 ± 0.02	1.27 ± 0.01	1.26 ± 0.02
6 g/kg	1.27 ± 0.01	1.29 ± 0.01	1.27 ± 0.02
12 g/kg	1.34 ± 0.01**	1.29 ± 0.01	1.30 ± 0.01

Parameters determined from blood collected at 24, 48, or 72 h post-EG treatment. Data are represented as means ± SEM (n = 6 for 24 and 72 h; n = 8 for 48 h). Asterisks (*) indicates significant difference from 0 g/kg control as determined by one-way ANOVA followed by Dunnett’s *post hoc* test, **p < 0.01, ***p < 0.001. HCO_3_^-^, bicarbonate; iCa^2+^, ionized calcium

### 3.3 Ethylene glycol (12 g/kg) significantly increases serum creatinine in male CD-1 mice at 72 h

Only the two highest doses of ethylene glycol (6 and 12 g/kg) induce significant increases in serum creatinine at 72 h **(**Fig. 2**C)**. No significant increases in blood urea nitrogen (BUN) **(**Fig. 2**D-F)**, calcium oxalate tissue accumulation, or overt renal histopathological damage were observed when compared to 0 g/kg treated mice at each time point. A significant decrease in BUN was observed in mice treated with 12 g/kg EG at 48 h compared to 0 g/kg treated mice. This was likely induced by an EG-mediated, interstitial dilutional effect **(**Fig. 2**E)**, but is not observed at 24 **(**Fig. 2**D)** or 72 h **(**Fig. 2**F)**. Furthermore, there were no significant increases or decreases in kidney to body weight ratios at any time point nor at any of the EG doses as mentioned previously.

### 3.4 Ethylene glycol (12 g/kg) induces proximal tubule dilatation in male CD-1 mice at 72 h with no calcium oxalate deposition

**Fig. 3:**
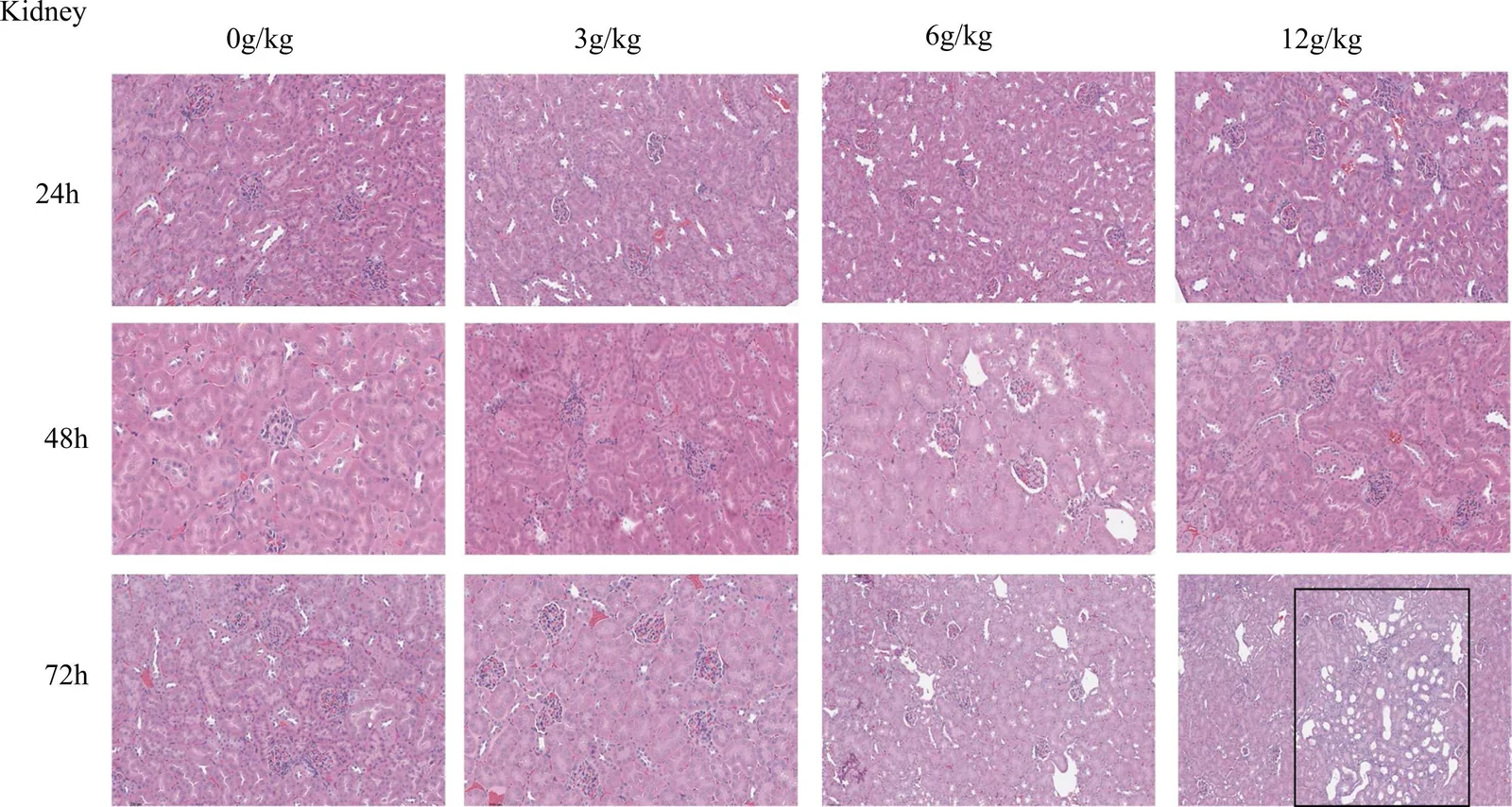
Representative hematoxylin and eosin images at 24, 48, and 72 h of cortical renal tissue from male CD-1 mice within each treatment show minimal to no damage for mice treated with 0, 3, or 6 g/kg EG, but moderate to severe kidney injury without calcium oxalate deposition for mice treated with 12 g/kg EG at 72 h. Magnification x 20x for all images.

**Fig. 4:**
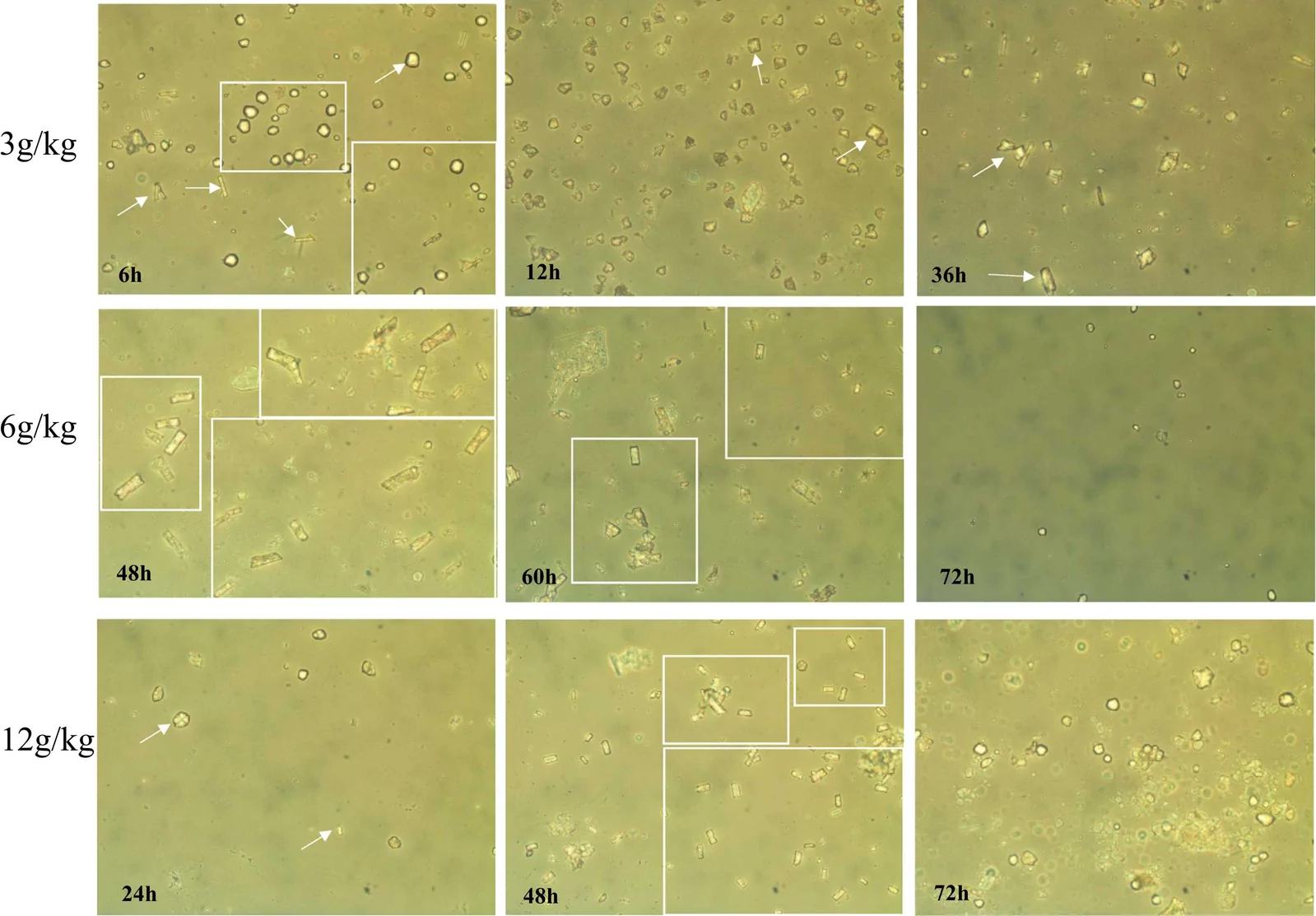
Representative images at various timepoints of calcium oxalate crystal formation from male CD-1 mice treated with 3, 6, or 12 g/kg EG. White arrows indicate specific calcium oxalate crystals; white boxes indicate collections of calcium oxalate crystals. Magnification x 20x for all images.

**Fig. 5:**
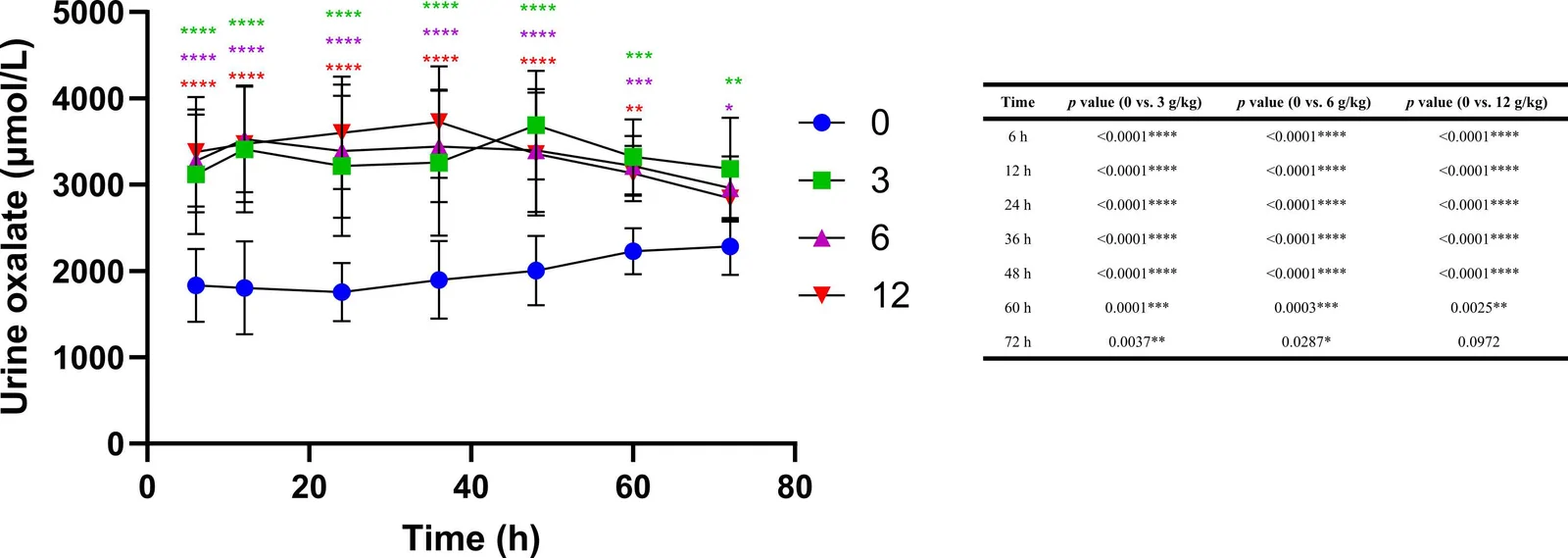
Acute ethylene glycol (3, 6, or 12 g/kg) administration at 24, 48, or 72 h in male CD-1 mice induces significant increases in urinary oxalate concentrations at 6, 12, 24, 36, 48, 60, and 72 h with no significant urine oxalate detected at 72 h in animals receiving 12 g/kg EG. Data is represented by mean ± S.E.M. (*n* = 6–8). Asterisks (*) indicate significant difference when compared to 0 g/kg EG control animals at each time point, and was analyzed using a one-way ANOVA followed by a Dunnett’s *post-hoc* test. *p < 0.05; **p < 0.01, ***p < 0.001, ****p < 0.0001 with individual *p* values indicated in the corresponding Table.

**Fig. 6:**
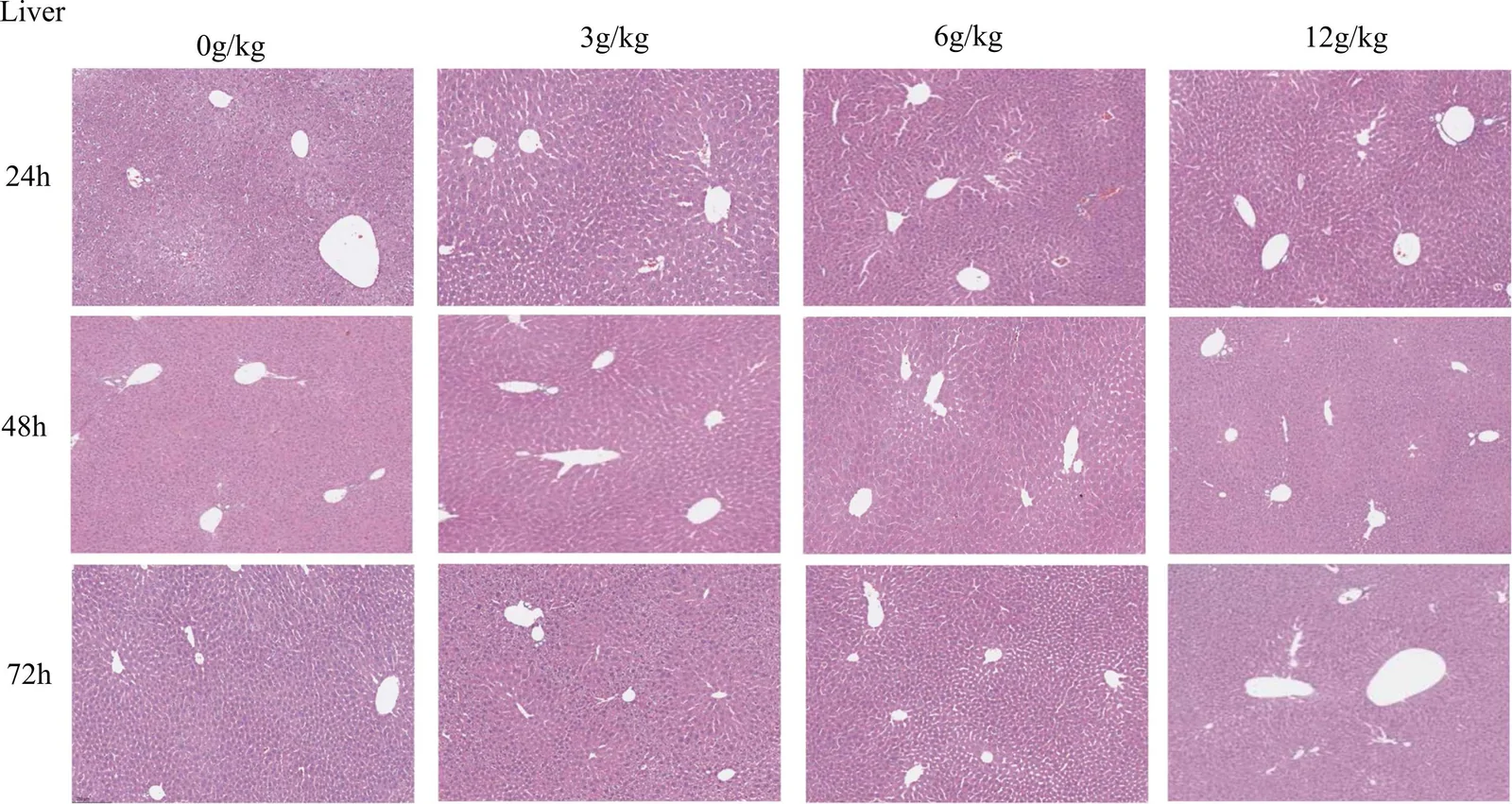
Representative hematoxylin and eosin images at 24, 48, and 72 h of hepatic tissue from male CD-1 mice within each treatment show no significant damage indicated for mice treated with 0, 3, 6, or 12 g/kg EG. Magnification x 20x for all images.

**Fig. 7:**
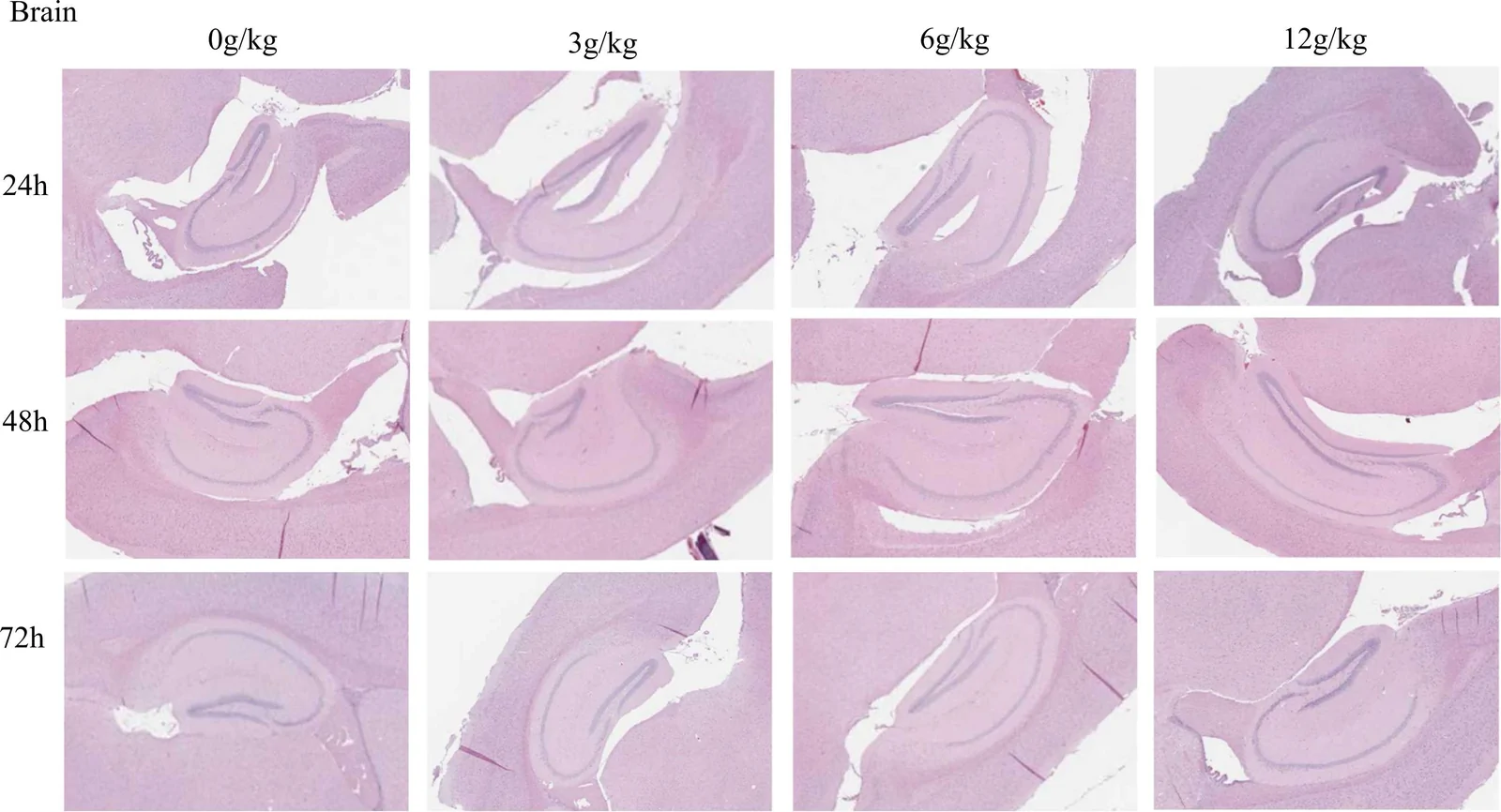
Representative hematoxylin and eosin images at 24, 48, and 72 h of hippocampal tissue from brain slices of male CD-1 mice within each treatment show no significant damage indicated for mice treated with 0, 3, 6, or 12 g/kg EG. Magnification x 20x for all images.

Only the high dose of ethylene glycol (12 g/kg) induced renal proximal tubule dilatation at 72 h **(**Fig. 3**).** No tissue calcium oxalate deposition nor significant renal histopathological changes occurred at 3 or 6 g/kg EG at 24, 48, or 72 h **(**Fig. 3**)**. However, urinary analysis revealed significant calcium oxalate crystal excretion across representative time points **(**Fig. 4**)** with significant increases in urinary oxalate concentrations reaching 3–4 mmol/L in animals 6, 12, 24, 36, 48, and 60 h post- 3, 6, and 12 g/kg EG administration when compared to 0 g/kg EG controls **(**Fig. 5**)**. Animals administered 12 g/kg EG did not have significant urinary oxalate concentrations at 72 h, unlike the 3 and 6 g/kg EG groups. Furthermore, 3, 6, or 12 g/kg EG did not induce significant histopathological changes in the liver **(**Fig. 6**)**. Only marginal to minimal vacuolation was reported in the brain (hippocampus, specifically) of treated male CD-1 mice after 24, 48, or 72 h of exposure **(**Fig. 7**)**. Furthermore, histopathology scores supported moderate EG-induced renal tissue damage with only minimal brain vacuolation, and no significant changes in liver histopathology **(**Table 2**)**.

**Table 2:** Kidney and brain histopathology scores in EG treated CD-1 male mice.

Kidney				Brain	
	Tubular dilatation	Inflammation	Necrosis		Vacuolation
Treatment Group					
0 g/kg	**0**	**0**	**0**		**0**
3 g/kg	**1** (n = 1); **2** (n = 2)	**1** (n = 1)	**0**		**1** (n = 3)
6 g/kg	**1** (n = 2); **2** (n = 1)	**1** (n = 1)	**1** (n = 1); **2** (n = 2)		**1** (n = 2)
12 g/kg	**2** (n = 1); **3** (n = 2)	**1** (n = 1)	**0**		**1** (n = 3)

Data are represented as the severity scores in **bold** included in the Materials and Methods with the number of

animals reported to possess histopathological abnormality/per group in parentheses (n = 6–8). Only the histopathological

assessments with scores are included in this Table. All other assessments were negative (0).

### 3.5 Ethylene glycol does not increase brain inflammation in male CD-1 mice even at high doses

**Fig. 8:**
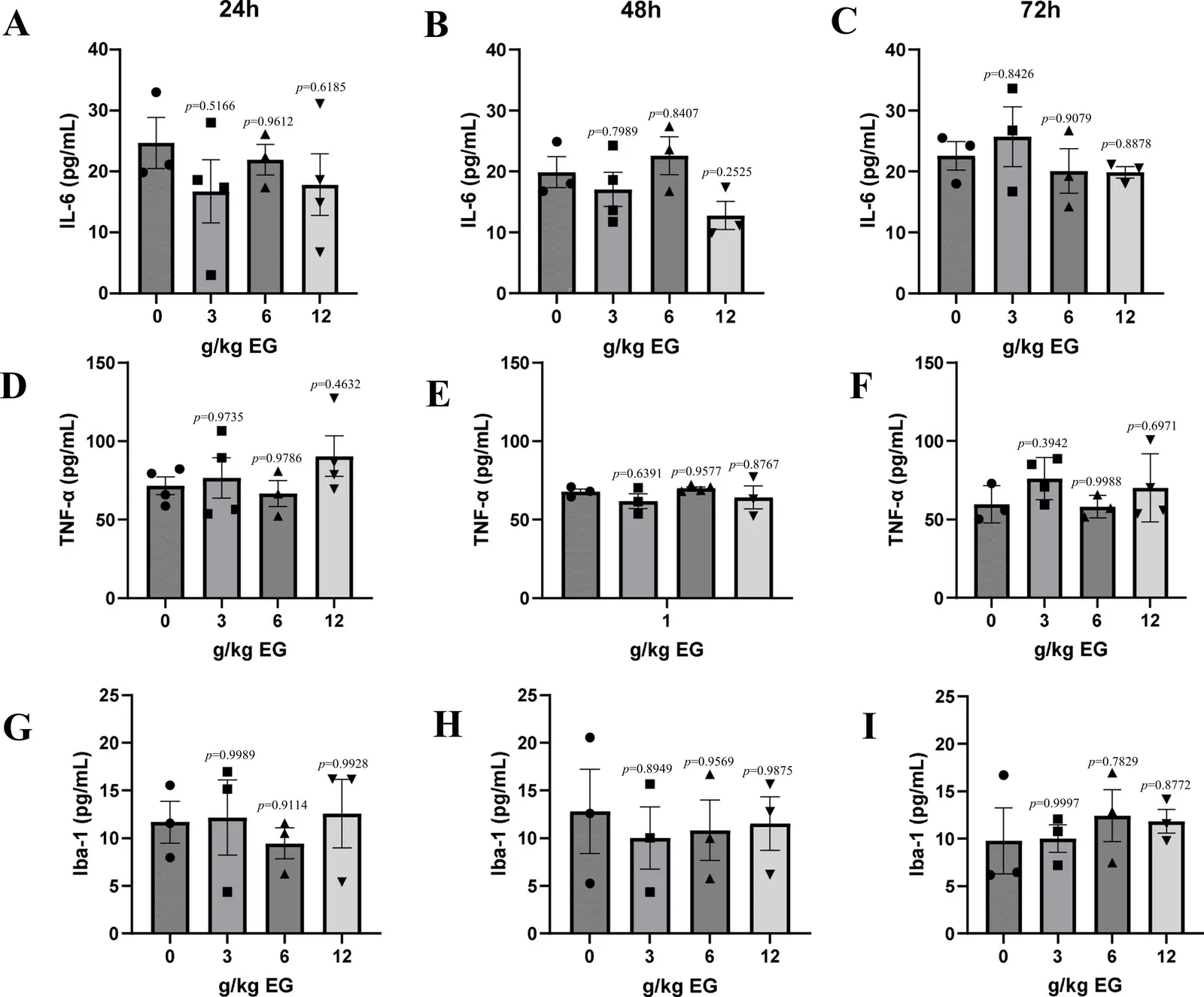
Acute ethylene glycol (3, 6, or 12 g/kg) administration at 24, 48, or 72 h in male CD-1 mice does not induce significant increases in specific brain tissue inflammatory markers: IL-6 (A-C), TNF-α (D-F), or Iba-1 (G-I). Data is represented by mean ± S.E.M. (*n* = 3–4), and was analyzed using a one-way ANOVA followed by a Dunnett’s *post-hoc* test; p < 0.05 would have indicated significance when compared to 0 g/kg EG at each time point with individual *p* values indicated above each EG treatment group.

Ethylene glycol does not significantly increase major brain inflammatory markers: IL-6 **(**Fig. 8**A-C)**, TNF-α **(**Fig. 8**D-F)**, or Iba-1 **(**Fig. 8**G-I)** in male CD-1 mice at 3, 6, or 12 g/kg at 24, 48, or 72 h indicative of no acute neuroinflammation even at high doses.

### 3.6 Oxalate ions induce cytotoxicity in mouse TKPTS proximal tubule cells in vitro

**Fig. 9:**
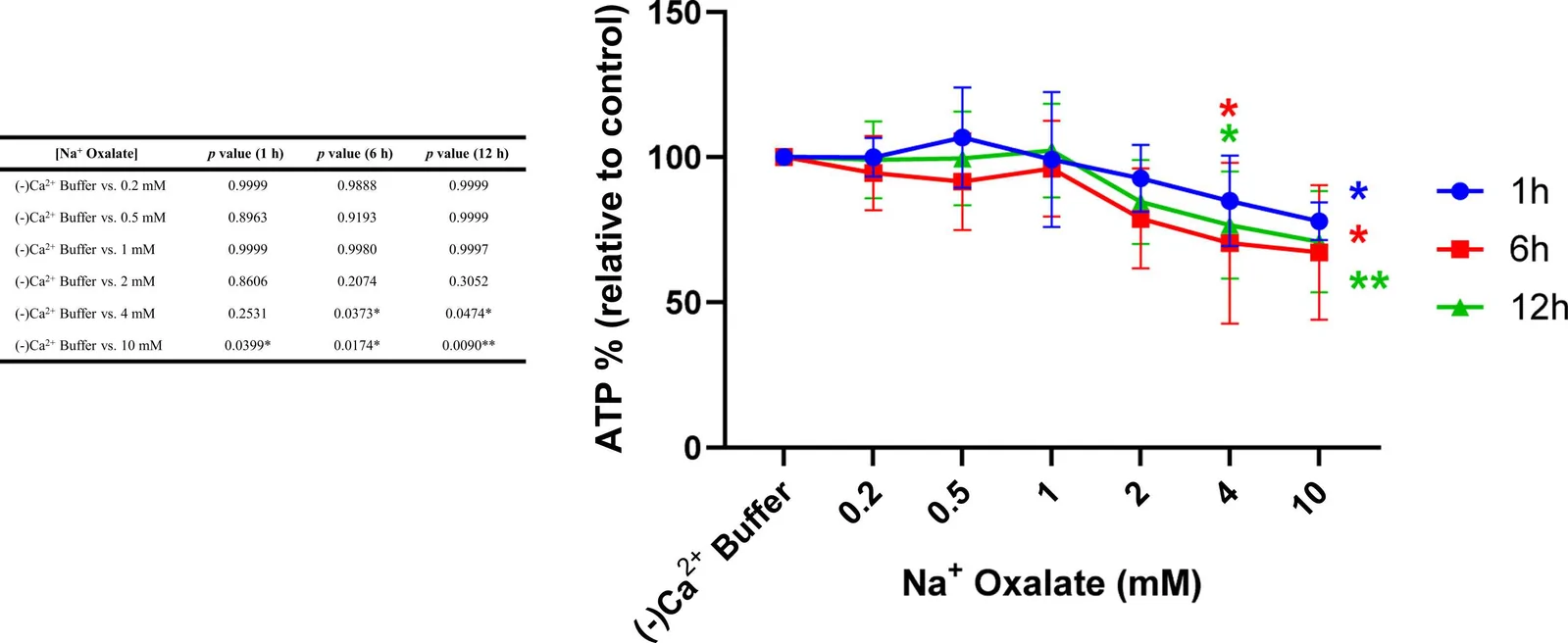
Sodium (Na^+^) oxalate alone in calcium free ((-)Ca^2+^) buffer induces significant decreases in ATP concentrations in mouse TKPTS proximal tubule cells *in vitro* at 4 mM for 6 and 12 h and at 10 mM for 1, 6, and 12 h. Data is represented by mean ± S.E.M. (*n* = 6). Asterisks (*) indicate significant difference when compared to (-)Ca^2+^ buffer controls, and was analyzed using a one-way ANOVA followed by a Dunnett’s *post-hoc* test. *p < 0.05; **p < 0.01 with individual *p* values indicated in the corresponding Table.

The oxalate ion alone induces significant decreases in mouse TKPTS proximal tubule cell ATP levels at 4 mM after 6 and 12 h of exposure, and at 10 mM concentrations after 1, 6, and 12 h of exposure (Fig. 9**)** indicating a non-calcium oxalate monohydrate (COM) crystal-mediated mechanism of mouse kidney damage post-EG administration.

## 4 Discussion

Multiple studies have characterized the toxicity of EG across a wide range of species including rats, rabbits, B6C3F_1_, DDG, and female CD-1 mice. However, the vast majority of studies assessing EG toxicity in male CD-1 mice have primarily, if not solely, focused on the developmental and reproductive toxicity of EG (Table 3) [11,13,16,17,20,21,[24], [25], [26], [27],^45^,^46^]. One recent study characterized acute EG toxicity at the reported LD_50_ in male DDG mice at 24 h reporting significant kidney toxicity characterized by oliguria, renal tubular cell degeneration, necrosis, and infiltration of inflammatory cells in the renal tubules [11]. However, no other EG-target organs were assessed at later time points. Furthermore, rodent species and strain, for example Wistar vs. Fischer-344 rats, have been reported to significantly affect EG sensitivity due to differences in protective urinary protein expression. These differences ultimately affect calcium oxalate super-saturation allowing for greater calcium oxalate monohydrate (COM) deposition in male Wistar rats when compared to male Fischer-344 rats [47,48]. Interestingly, male CD-1 mice in our studies showed no COM crystal accumulation in renal tissue also supporting previous evidence that CD-1 mice are not susceptible to nephrocalcinosis induced by dietary 50µmol/g sodium oxalate fed over a period of 21 days [49]. Our results indicate this is due to rapid calcium oxalate urinary excretion in this mouse strain as observed across representative time points in Fig. 4, and across all time points in Fig. 5. Additionally, differences in alcohol dehydrogenase expression in CD-1 male mice could also explain decreases in EG target organ toxicity; however, previous literature indicates a similar expression pattern and enzyme activity to that of humans [[50], [51], [52]]. At 72 h post EG-gavage, mice in the 6 and 12 g/kg EG dose groups had significantly increased serum creatinine levels, but no visible COM accumulation in the kidney tissues explained by a high crystal excretion rate in mice and direct oxalate ion-induced kidney toxicity. Previous literature indicates the oxalate ion (potassium oxalate) can induce significant decreases in rat kidney cell viability after 18 h of exposure at concentrations as low as 500 µM, as well as induce significant oxidative stress and endoplasmic reticulum dysfunction in the kidneys of rats fed 5% potassium oxalate [53,54]. Additionally, in isolated mouse proximal tubule segments from male CD-1 mice, oxalate alone caused no significant lactate dehydrogenase (LDH) release, nor it induce any increases in lipid peroxidation after 1 h of exposure [3]. It is well known that the proximal tubule is the primary target of COM crystal-induced renal toxicity in primary human and rat isolated proximal tubule cells *in vitro* [[5], [6], [7], [8], [9],^47^,^48^]. Previous studies have also assessed oxalate-induced toxicity in mouse inner medullary collecting (mIMCD-3) cells with 1, 2, 4, and 10 mM concentrations inducing a decrease in cell density, and a subsequent increase in membrane permeability [55,56]. However, these studies were not completed in a calcium-free environment; therefore, oxalate ion or COM-induced mIMCD-3 cell toxicity remained inconclusive.

**Table 3:** Strain, sex, dose, route of exposure (RoE), exposure duration, & toxicity assessment variation in major mouse EG studies.

**Mouse Strain/ Sex**	**Dose(s) / RoE**	**Duration**	**Organ(s) Toxicity Reported**	**Reference**
ICR Swiss/ F	0.03 mL/g / i.p.	Single dose	LD_50_ + /- alkyldiol antidotes	Holman, *et al.,* 1979
B6C3F_1_/ M, F	0.32–5% / diet	91 days	LD_50_, kidney (M only), testes	Melnick, *et al.,* 1984
CD-1/ F	0.75–3 g/kg/day / p.o.	15 days	Minimal teratogenicity	Price, *et al.,* 1985
CD-1/ M, F	0.25–5% / drinking water	15 days	Minimal teratogenicity	Lamb, *et al.,* 1985
B6C3F_1_/ M, F	0.2–1 g/kg/day / p.o.	4 days	Bone marrow (M/F), seminiferous tubules	Hong, *et al.,* 1988
CD-1/ M, F	0.05–2.5 g/m^3^ / inh.	18 days	Minimal teratogenicity	Tyl*, et al.,* 1995
CD-1/ F	0.05–1.5 g/kg / p.o.	15 days	NOAEL for teratogenicity	Neeper-Bradley, *et al.,* 1995
CD-1/ M, F	0.5–2.5 g/m^3^ / nose inh.	18 days	Minimal teratogenicity	Tyl, *et al.,* 1995
CD-1/ F	0.1–0.4 g/kg / p.o. 0.1–1 g/kg / i.v. 1 g/kg / s.c.	4 days	EG tissue distribution, dose-dependent urinary elimination (EG, metabolites); no toxicity	Frantz, *et al.,* 1996
Black Swiss/ M	1% / drinking water	28 days	Hyperoxaluria, nephrolithiasis	Wesson, *et al.,* 2003
C57BL/6/ M, F	1.25% / diet	28 days	Kidney, COM deposition	Khan & Glenton, 2012
ddY/ M	0.0005–15 g/kg / p.o.	Single dose, 1 day	LD_50_, kidney	Meles, *et al.,* 2024

NOAEL, No Observed Adverse Effect Level; COM, Calcium oxalate monohydrate; i.p., intraperitoneal; p.o., per oral (gavage); inh., inhalation; nose inh., nose-only inhalation; i.v., intravenous; s.c., subcutaneous; ddY, Deutschland Denken & Yoken

It is likely that the kidney injury observed in our mice at 72 h could be a result of oxalate alone, and not the toxicity of COM Interestingly, at 72 h mice administered 12 g/kg EG did not have significant urinary oxalate excretion, which could be explained by decreases in renal function as evident by the observed significant increases in serum creatinine. Furthermore, our *in vitro* studies in mouse TKPTS proximal tubule cells indicate that the oxalate ion alone at 4 and 10 mM does induce significant decreases in cellular ATP concentrations at 6 and 12 h and 1, 6, and 12 h, respectively. Such significant decreases in ATP would result in significant proximal tubular epithelial cell injury. This could further explain why there were significant increases in serum creatinine levels, proximal tubule dilatation, and decreased oxalate excretion in the 12 g/kg animals at 72 h. Thus, oxalate ion-induced toxicity in the mouse proximal tubule early on likely explains the observed kidney damage at 72 h. According to the literature, this is the first time that oxalate ion-induced toxicity has been assessed in TKPTS mouse proximal tubule cells *in vitro*. One minor limitation is that earlier time points were assessed in our *in vitro* studies to decrease any off-target toxic effects that culturing cells in a calcium free environment would elicit as to not confound the results or conclusions [57]. Additionally, mechanisms explaining oxalate-induced decreases in ATP in our *in vitro* studies, such as oxidative stress, mitochondrial function, and inflammatory pathways would need to be addressed in future studies Interestingly, it was also observed that at 48 h post-EG administration, animals dosed with 12 g/kg EG exhibited a significant decrease in BUN, which then rectified by 72 h and could likely be explained by animals in this dose group experiencing a large volume, diluent effect post-EG gavage.

It is well-known that the alcohol-like properties of EG contribute to inebriation early in the course of toxicity with the potential for CNS depression and delayed neurological sequelae with accompanying cranial nerve deficits observed post-EG induced AKI [[58], [59], [60]]. Cranial nerve deficit etiology is hypothesized to be due to COM deposition within the cerebral vasculature or ethylene glycol-mediated pyridoxine dysfunction [60,61]. Previous case reports of ethylene glycol intoxication in humans report delayed neurological dysfunction 3–20 days post-ingestion at which time ethylene glycol has been fully metabolized to its toxic metabolites [29]. The vast majority of neurological dysfunction in these cases involve cranial nerves II, V, VIII, IX, X, and XII with 70.8% patient recovery. The volume of distribution (*V*_*d*_*)* of EG in humans is approximately 0.5–0.83 L/kg which is relatively low, and indicative of low CNS distribution [62]. This further supports the necessity of assessing these EG-induced CNS effects at these high EG doses in a very commonly utilized mouse strain. In our studies, there was no COM crystal deposition in brain tissue, and no significant EG-induced gross neurohistology changes observed at the reported (6 g/kg) and 2-fold the reported (12 g/kg) toxic doses in our mice. This was supported by unremarkable gross, brain histopathology, the ability of the animals to maintain healthy body weight, as well as no significant differences in the brain:body weight ratios of animals treated with EG at any of the time points assessed. Additionally, as EG-induced brain inflammation could also likely play a significant role in EG-mediated neurotoxicity, two major inflammatory cytokine levels and a major marker of microglial activation (IL-6, TNF-α, and Iba-1, respectively) were assessed in brain tissue from all EG-treated groups at all time points, and compared to the 0 g/kg controls. Our results indicate that EG-treated animals had no significant increases in brain IL-6 or TNF-α levels, nor did they have significant increases in microglial infiltration or activation. This further supports the likelihood that the CNS is not a major target organ of EG toxicity. Furthermore, there were no COM crystals observed in the brain tissue of EG treated animals, which was not surprising as COM is associated with direct endothelial cell toxicity, and likely does not significantly cross the blood brain barrier to directly damage neuronal tissue [4]. As mentioned previously, the lack of target organ COM accumulation could also be explained by the significant increases in urinary COM and oxalate in these mice, and the lack of nephrotoxicity neurohistological abnormalities, and neuroinflammation could be due to species-specific differences in renal elimination. Furthermore, we can conclude that male CD-1 mice are significantly resistant to EG at these high doses, which could provide a strain to model large EG ingestions observed in humans.

In conclusion, our studies indicate that high doses of EG at acute time points induce significant, albeit transient, metabolic acidosis, high urinary oxalate excretion coupled with minimal oxalate ion-induced nephrotoxicity, and no significant neurohistological abnormalities or neuroinflammation in male CD-1 mice. Future studies would assess mechanisms of oxalate ion mediated nephrotoxicity, potentially provide this strain as a model for the assessment of nuanced neurological changes at high EG doses, as well as the variability in the handling of EG across sensitive and non-sensitive strains and species.

## CRediT authorship contribution statement

**Greg M. Landry:** Writing – review & editing, Writing – original draft, Visualization, Validation, Supervision, Project administration, Methodology, Investigation, Funding acquisition, Formal analysis, Conceptualization. **Thomas Q. Dong:** Project administration, Methodology, Investigation, Formal analysis, Data curation. **James Boksanski:** Resources, Methodology, Formal analysis, Data curation.

## Funding

This research was supported by the Ethylene Glycol/Ethylene Oxide Panel of the 10.13039/100007824American Chemistry Council (ACC). Instrumentation was provided by the Massachusetts Life Sciences Center: Workforce Development Capital Grants.

## Declaration of Competing Interest

The authors declare the following financial interests/personal relationships which may be considered as potential competing interests: Thomas Q. Dong reports financial support was provided by American Chemistry Council Inc. If there are other authors, they declare that they have no known competing financial interests or personal relationships that could have appeared to influence the work reported in this paper.

## Data Availability

Data will be made available on request.
